# Video-based on-ward supervision for final year medical students

**DOI:** 10.1186/s12909-015-0430-2

**Published:** 2015-12-01

**Authors:** JB Groener, TJ Bugaj, R. Scarpone, A. Koechel, J. Stiepak, S. Branchereau, M. Krautter, W. Herzog, C. Nikendei

**Affiliations:** 1 Department of Endocrinology and Clinical Chemistry, University of Heidelberg, Heidelberg, Germany; 2 Department of General Internal and Psychosomatic Medicine, University of Heidelberg, Heidelberg, Germany; 3 Department of Cardiology, Angiology, Pneumology, University of Heidelberg, Heidelberg, Germany; 4 Department of Nephrology, University of Heidelberg, Heidelberg, Germany

**Keywords:** Medical Student, Real Patient, Workplace Learning, Ward Round, Procedural Skill

## Abstract

**Background:**

Constructive feedback is an essential element of the educational process, helping trainees reach their maximum potential and increasing their skill level. Video-based feedback has been described as highly effective in various educational contexts. The present study aimed to evaluate the feasibility and acceptability of video-based, on-ward supervision for final year students in a clinical context with real patients.

**Methods:**

Nine final year medical students (three male, six female; aged 25.1 ± 0.7 years) and eight patients (five male, three female; aged 59.3 ± 16.8 years) participated in the pilot study. Final year students performed routine medical procedures at bedside on internal medicine wards at the University of Heidelberg Medical Hospital. Students were filmed and were under supervision. After performing the procedures, an oral feedback loop was established including student, patient and supervisor feedback on communicative and procedural aspects of skills performed. Finally, students watched their video, focusing on specific teachable moments mentioned by the supervisor. Written evaluations and semi-structured interviews were conducted that focused on the benefits of video-based, on-ward supervision. Interviews were analysed qualitatively, using open coding to establish recurring themes and overarching categories to describe patients’ and students’ impressions. Descriptive, quantitative analysis was used for questionnaire data.

**Results:**

Supervised, self-chosen skills included history taking (*n* = 6), physical examination (*n* = 1), IV cannulation (*n* = 1), and ECG recording (*n* = 1). The video-based, on-ward supervision was well accepted by patients and students. Supervisor feedback was rated as highly beneficial, with the video material providing an additional opportunity to focus on crucial aspects and to further validate the supervisor’s feedback. Students felt the video material would be less beneficial without the supervisor’s feedback. The setting was rated as realistic, with filming not influencing behaviour.

**Conclusion:**

Video-based, on-ward supervision may be a powerful tool for improving clinical medical education. However, it should be regarded as an additional tool in combination with supervisors’ oral feedback. Acceptance was high in both students and patients. Further research should address possibilities of efficiently combining and routinely establishing these forms of feedback in medical education.

## Background

To ensure a smooth transition from university to clinical practice, workplace learning is of the utmost importance [1, 2]. In Germany, the final year of medical education comprises three 4-month clerkships in medical specialties (internal medicine, surgery and a third elective subject), serving to integrate final year medical students into their future working environment [3, 4]. Final year students are expected to assist medical doctors with their daily on-ward routines, learning to admit patients, handle medical cases, manage ward rounds and perform routine procedures such as IV cannulation, drawing blood, or recording ECGs. In terms of these ambitious educational objectives, workplace learning represents a challenging facet of undergraduate education. Various educational interventions have been introduced to enhance the didactic value of workplace learning, for example, introductory courses [5], accompanying seminars [6], logbooks [7] and portfolios [8]. However, international observations have highlighted that workplace learning during clerkship assignments and final year education still shows severe deficits, with a lack of structure, integration, supervision and personal feedback [9–12].

Some innovative models have begun to emerge to address these limitations in final year medical education, for example, establishing educational wards offering the supervised treatment of real patients [13–16]. However, these approaches are often costly and require considerable resources and expertise. Therefore, we investigated an innovative model for structured, on-ward supervision of final-year students [17].

To acquire clinical competencies, feedback is seen as a central factor supporting individual learning processes [18]. Here, informational as well as motivational feedback factors represent potent stimuli for behaviour modification (see [19] for a comprehensive review). Nevertheless, the correct form of feedback delivery is still strongly debated (for example quantity vs. repetition) [20–24]. However, the fact that the quality of feedback has a significant impact on objective training success remains undisputed [25–28]. In terms of different forms of feedback, video-feedback has been found to be highly effective, as shown in the acquisition of resuscitation skills [29, 30] and surgical techniques [31–33]. In a recently published study on oral presentations of clinical cases publicly presented by medical students, video-assisted, oral feedback reduced severe anxiety during presentations when compared with ‘usual practice’ [34]. However, it remains unclear whether it is feasible to integrate video-feedback into clinical internal medicine routines in the ward setting and if such an innovative model would be accepted.

### Aims of the present study

While the overall aim of the present study was to develop and establish a video-based, on-ward supervision model, our study sought to (1) evaluate the feasibility and acceptability of video-based, on-ward supervision via the assessment of process, resources, management and scientific factors; and, (2) assess whether video-based, on-ward supervision was perceived as beneficial by participating final year students.

### Conceptual framework

We used Ericsson’s model of deliberate practice as the conceptual framework for our approach [35]. Ericsson characterised training as a highly structured activity, directed at improving performance in a particular domain [36]. Therefore, deliberate practice can be understood to be a focused approach to training, aiming to reach a well-defined goal [37]. Practical implementation of this construct was based on several design principles [38], one of which is specific, informative feedback, which can ultimately lead to sustainable behaviour modification [35]. Video-based feedback can therefore be seen as a training element useful to achieve certain goals.

## Methods

### Study design

The present study was a pilot study using a mixed-method approach [39], involving the systematic integration of quantitative and qualitative methods to obtain a complete picture [40]. We focused on feasibility, implementation issues and participants’ quantitative and qualitative perceptions of potential benefits of video-based, on-ward supervision. We used a minimally structured and open interview style to record a variety of impressions and perceptions. There were no pre-defined categories reflecting these methodological approaches [41]. For the qualitative parts of our study we used a grounded theory design, as this is detailed and systematic and therefore suitable to investigate complex phenomena with a focus on interactions in specific situations [42]. Our innovative model was developed by an expert team (CN, JG, TB, JS, AK), based on a previously published, on-ward supervision programme [17].

### Participants

In German medical school programmes, the sixth year is also known as the final year. Final year students must complete one term of internal medicine, one term of surgery and one elective term (that is a 4-month period of on-ward training in each specialty). All final year students working in internal medicine departments at the University of Heidelberg Medical Hospital between December 2013 and March 2014, were invited to participate in video-based, on-ward supervision by e-mail and during lectures. Students were informed that participation was voluntary, and that nonparticipation would have no influence on other aspects of their final year medical programme or grades.

One experienced supervising doctor, a 4th year internal medicine resident (JG), specially trained in medical student education and who was not the supervising ward physician, observed all participating students’ patient admissions and performance of procedural skills at bedside. Following this observation, the supervising doctor provided the students with structured feedback following a supervisor manual based on methods by Roos et al. [43].

All patients were internal medicine inpatients at the University of Heidelberg Medical Hospital. Preferably, participating patients were located on the ward in which the student worked.

### Setting

The procedure of the present study is presented in Table 1. Video-based, on-ward supervision with real patients was performed once with each participating final year student. The sessions were held on the medical wards of the University of Heidelberg Medical Hospital in which the participating student worked (nephrology, cardiology, and endocrinology subspecialty wards). Participating students self-selected the procedure to be supervised, depending on patient availability, personal interest and self-perceived supervision needs. Skills selected were: 1) history taking (*n* = 6); 2) a complete internist’s physical examination (*n* = 1); 3) IV cannulation (*n* = 1); and, 4) performing an ECG recording (*n* = 1). When possible, patients were initially invited to participate by the student and in exceptional circumstances by the responsible supervisor (for example the student was not working on a ward with sustainable patient contacts, such as in the emergency room). Supervision was conducted in the patient’s room on the ward. Patients with language difficulties, dementia, or psychological instability were excluded from participation.

**Table 1:** Procedure for video-based, on-ward supervision and time needed for specific parts of the session

Procedure	Time needed [min]
First talk with students	10 min
Getting patient’s and student’s informed consent	5–10 min
Setting up equipment	5 min
Supervision with video-taping	15–20 min (depending on supervised skill)
Feedback loop	5 (−10) min
Watching the video	15–20 min (as long as video runs)
Filling out evaluation forms	2–3 min
Interview	7–15 min

#### Supervisor’s instructions and student competencies

The supervisor was specifically trained in the supervision of final year medical students during clinical on-ward procedures and to provide professional, structured feedback. During supervision, the supervisor provided students with standardised instructions and outlined expectations, for example, the respectful treatment of patients during all contact. For history taking, a complete history of the patient was expected following the standard University of Heidelberg procedure [44]. Students who chose to be supervised during a physical examination were required to perform a complete physical examination according to the Heidelberg standard for patient admission on the internal medicine ward (pupils, oral cavity, lymph nodes, thyroid gland, heart, pulse, lungs, abdomen, vertebra, muscular strength and sensitivity) [45]. They were instructed to perform the required skills as if no supervisor was present (that is no oral explanations of what they were doing other than communication with the patient). For the ECG recording, following pre-existing checklists [44], and interpretation was expected. However, only the recording procedure was supervised in the video-based approach, whereas the interpretation was supervised outside the patient’s room and was not filmed. For IV cannulation, the student was expected to arrange required material and perform the procedure following a pre-existing checklist [44]. Students’ personal notes during history taking or physical examination were not assessed.

#### Supervisor’s video recording and field notes

A Rollei Movieline SD-23 (Rollei GmbH & Co. KG, Hamburg, Germany) camera was used to record the performance of clinical skills. It was placed in the corner of the patient’s room facing the patient’s bed, without showing a neighbouring patient in the same room. The camera was operated by the chief investigator (JG), with occasional assistance by a second team member (RS). The camera position was only adapted during the supervision of manual skills (for example the use of the camera zoom during IV cannulation) to show some aspects of the skill in more detail. Field notes were taken by the supervisor during each session to record relevant positive or improvable procedural or communicative aspects. These aspects were then discussed fully in the feedback loop.

#### Feedback loop

After completion of the supervised procedure, a standardised feedback loop was performed. Feedback itself was not filmed. First, the student gave self-reflective feedback including the setting (seating position, distance between student and patient, avoidance of disturbances), and the procedure (technical and communication skills). This was followed by the patient’s oral feedback, addressing the setting and a subjective assessment of the student’s performance. Finally, the supervisor gave the student oral feedback regarding the setting, the student’s behaviour, and their performance of the technical skill. Most of the supervisor’s feedback was given in the patient’s room in their presence. If necessary, any major professional issues were addressed outside the patient’s room. Before watching the video, the supervisor identified specific issues requiring focus. A quiet room on the ward was chosen for students to view their videos. In most cases, viewing was undertaken in the presence of the supervisor, who did not comment during the video. After watching the video, the student was asked whether he/she was able to relate to the supervisor’s feedback and whether new aspects not initially identified had been observed.

### Data collection

#### Students’ quantitative assessment

For pre-assessment, a questionnaire was administered seeking information on baseline characteristics (for example age, sex, career aspirations) as well as assessing students familiarity with the required skills (history-taking, physical examination, patient presentation, IV cannulation) during their studies in a) controlled (skills lab, simulation, standardised patients) or b) real conditions. Finally, students were asked to self-assess each of the four skills regarding their feeling of preparedness for entry into their profession. Self-assessment was on a 7-point Likert scale (1 = Very untrue; 2 = Untrue; 3 = Somewhat untrue; 4 = Neutral; 5 = Somewhat true; 6 = True; 7 = Very true).

After watching the video, students completed an evaluation form specifically addressing the benefits of video-based supervision. Responses were on a 6-point Likert scale that deliberately avoided a neutral score; ensuring participants rated their intervention experience in terms of a ‘forced choice’. The questionnaire consisted of 13 items (statements about the benefit of the video-based supervision and the experienced feedback), rated individually on a scale from 1 to 6 (1 = fully disagree, 6 = fully agree).

#### Students’ qualitative assessment

The interview procedure followed the consolidated criteria for reporting qualitative research (COREQ) checklist [46] and the Standards for Reporting Qualitative Research (SQOR), as recently published [47]. The COREQ checklist was developed to promote comprehensive reporting of interviews and focus groups in qualitative studies. It has 32 criteria that can aid researchers in reporting important aspects of the research team, study methods, study context, findings, analysis and interpretations [46]. The 21-item SQOR defines standards for qualitative reporting including information on data collection, processing, analysis and limitations.

Individual, face-to-face interviews were conducted with all participating students. Interviews lasted approximately 20 min and were conducted by a trained interviewer who was supervised by an experienced tutor. The interviews were semi-structured [48–50] with open-ended questions, enabling students to talk about the benefits and specific aspects of video-based, on-ward supervision. Interviews were audio-taped and transcribed verbatim for interpretation, and non-verbal behaviours and subjective characteristics of the interview were recorded. The interviewer used open-ended questions [51] to ask the students about being videotaped, their perceptions of the supervision setting and realism, as well as the quality of the feedback from different sources.

#### Patients’ quantitative assessment

Patients were asked to provide feedback via a post-assessment questionnaire. This questionnaire used a similar structure to that administered to the students (statements rated from 1 to 6; 1 = fully disagree, 6 = fully agree), and comprised seven items addressing the patients’ feelings during the supervision, their willingness to participate in future and their opinion on how important video-based, on-ward supervision was for final year students.

### Qualitative data

The content analysis followed grounded theory principles [41]. First, open coding of all interviews was conducted to identify recurring topics. Specific sentences (or combinations of sentences) were identified as a code, representing the most elemental unit of meaning [41]. These codes were summarised as relevant themes for each participant, using MaxQDA software (2010 version, VERBI Software – Consult – Sozialforschung GmbH, Berlin). Recurrent themes across different participants were compared and adapted until overarching categories could be defined. The assignment of codes to specific themes was conducted by two independent analysts, discussed to reach consensus and adjusted if necessary. In the final step, themes were consolidated into four relevant categories.

### Quantitative data

Descriptive quantitative data were managed with Microsoft Excel 2010 (Microsoft Deutschland GmbH, Unterschleissheim, Germany) and presented as mean ± standard deviation (SD) and median with interquartile range as applicable. A 7-point Likert scale was used to evaluate students’ abilities before the video-based supervision and a 6-point Likert scale was used post-supervision to assess patients’ and students’ opinions of the supervision.

### Ethics

We adopted ethical principles according to the 2013 World Medical Association Declaration of Helsinki: ethical principles for medical research involving human subjects. The study protocol was approved by the Ethics Committee of the University of Heidelberg. Students’ refusal to participate had no impact on subsequent evaluations or other assessments in the curriculum. Patients were advised they could refuse to participate without having to provide a reason or fear negative effects. Final year medical students and patients who were willing to participate gave written informed consent before participating in the study.

## Results

### Participant characteristics

#### Students

Nine final year medical students (56 % of all final year students invited) consented to participate in the present pilot study on a voluntary basis (3 male, 6 female; aged 25.1 ± 0.7 years). Eight students had studied medicine at the University of Heidelberg, and one at the University of Mainz. All participating students had previous experience of being filmed during their studies as part of communication training with standardised patients at our faculty [52].

#### Patients

Eight internal medicine patients consented to participate in the study (five male, three female; aged 59.3 ± 16.8 years). One patient was willing to participate twice, meaning one participating student took the patient’s history and another student performed a physical examination on the same patient an hour later.

### Students’ self-rated baseline characteristics

Students self-reported feeling most confident about placing peripheral intravenous portals, taking histories and executing a physical examination, whereas they self-reported being less confident about case presentations (Table 2).

**Table 2:** Students’ (*n* = 9) quantitative pre-assessment of self-reported confidence in clinical domains on a 7-point scale (1 = not true, 7 = entirely true)

How well do the following statements apply to you?	Median	Interquartile range
I feel well prepared for starting my career concerning history taking	6	5–6
I feel well prepared for starting my career concerning physical examination	6	5–6
I feel well prepared for starting my career concerning case presentation	5	4–5
I feel well prepared for starting my career concerning IV cannulation	6	6–6

### Students’ quantitative assessment

Acceptance ratings of the video-based, on-ward supervision are presented in Table 3. The model was well accepted by the participating students, leading to recommendations to other students, who subsequently volunteered to participate in future supervision. Overall, video-based, on-ward supervision was perceived as very beneficial for practical medical education (Table 3). Students found the video-based intervention more beneficial for technical than communicative skills. However, patients evaluated communication skills more highly than procedural skills. The supervisor’s feedback was generally seen as being beneficial to very beneficial, especially in regard to procedural skills. The medical ward setting was reported to be suitable for video-based, on-ward supervision, with neither students nor patients feeling discomfort while being filmed. Overall, students were willing to participate in more video-based, on-ward supervision in future.

**Table 3:** Students’ (*n* = 9) quantitative post-assessment of programme acceptance on a 6-point scale (1 = fully disagree, 6 = fully agree)

Question	Median	Interquartile range
The ward was appropriate for video-based on-ward supervision	6	6–6
I was ashamed to be filmed	1	1–2
The patient’s oral feedback helped me to better assess my communicative skills	4	3–4
The patient’s oral feedback helped me to better assess my technical skills	4	3–5
The supervisor’s oral feedback helped me to better assess my communicative skills	5	4–5
The supervisor’s oral feedback helped me to better assess my technical skills	5	5–6
Watching the video helped me to better assess my communicative skills	4	3–5
Watching the video helped me to better assess my technical skills	5	4–6
I will be able to perform the supervised skills better due to watching the video	5	4–6
I wish to have had video-based supervisions in other settings	4	3–4
Watching the video was helpful for my practical training	5	4–6
Video-based, on-ward supervision was helpful for my practical training	5	5–6
I would participate in video-based supervision again in the future	6	6–6

### Themes from student interviews

The qualitative analysis of the interviews covered four categories incorporating nine themes, as defined below (Table 4).

**Table 4:** Overview of categories and themes identified from interviews

Category 1: setting and realism of the situation	
Theme ‘Setting’	
Theme ‘Realism of the situation’	
Category 2: influence of being supervised and filmed	
Theme ‘Behavioural changes due to filming’	
Theme ‘Subjective feelings while being filmed’	
Category 3: students’ self-assessment	
Theme ‘Communicative skills’	
Theme ‘Procedural technical skills’	
Category 4: relevance of feedback	
Theme ‘Patient’s feedback’	
Theme ‘Supervisor’s feedback’	
Theme ‘Video-based feedback’	

#### Category 1: setting and realism of the situation (58 quotations)

##### Theme ‘Setting’ (16 quotations)

All students preferred a quiet environment without disturbances from telephones, incoming staff or visitors, stating ‘nobody entered the room, no one disturbed us; that was good’, and ‘[therefore] I was relaxed, and I had the feeling that the patient was relaxed as well’. One student also said that ‘switching the telephone [’s ring tone] off would have helped because [the] phone ringing [during history taking] distracts me’. Interaction with patients at eye level was regarded as optimal, and sitting next to each other was preferred to sitting opposite.

##### Theme ‘Realism of the situation’ (42 quotations)

As the intervention took place in patients’ rooms on medical wards, students found the context to be highly realistic: For example, ‘It felt like it usually feels when routinely performing an ECG recording in the same surroundings’. The supervisor’s choice of patients was considered to be more realistic than students choosing the patients themselves, as they would have had to first obtain permission (written informed consent was always obtained by the supervisor). One student noted that ‘the patient knew beforehand what my task was and that I am not his attending physician. That probably changed our relationship [that is to the patient], as the patient behaved more like an actor due to the more artificial situation’. The students preferred real patients to actors because ‘actors are fine, but by using real patients, you can draw a better comparison to how it really feels [to perform medical skills on a daily basis]’. Another student noted ‘an actual patient usually has a more profound story to tell than an actor, who specifically prepared for one situation by learning the symptoms by heart’, and ‘They [the actors] sometimes even help you’. Compared with working with a patient already admitted and simply repeating history taking, having to actually admit a patient on the ward also increased the realism of the situation: ‘you have to perform [the history taking] properly, otherwise you will have to go back to the patient to ask the questions you forgot, which I don’t like’.

#### Category 2: influence of being supervised and filmed (65 quotations)

##### Theme ‘Behavioural changes due to filming’ (32 quotations)

Students were familiar with the situation, having already experienced being filmed in previous classes. Most students stated they had generally performed skills according to their usual daily routine, but possibly with more diligence and dedication, for example, asking for more details during history taking owing to being filmed. It was agreed that being supervised might have also influenced their behaviour, regardless of the camera. One student commented ‘I made more comments [than usual] for the camera [while doing the physical examination], which were of no interest for the patient’. However, other students felt quite the opposite with the camera running, reporting they were less communicative and more focused on important subjects. A student stated critically that he thought ‘it is very difficult to be unbiased whilst videotaping, especially when it comes to behaviour [which will be different owing to being filmed]’ , and another commented ‘It felt 80 % realistic, but you had more time than usual for performing the skills’.

##### Theme ‘Subjective feelings while being filmed’ (33 quotations)

As students were aware of being filmed, they were able to cope with and largely ignore the presence of the supervisor and camera. One student noted that she ‘realised [she] occasionally looked into the camera instead of facing the patient’. However, not all students liked the feeling of being filmed, possibly experiencing a sense of embarrassment when later observing their behaviour on film. Another student was indirectly distracted by the camera, as the patient kept looking into the camera instead of talking to her during history taking.

#### Category 3: students’ self-assessment (20 quotations)

##### Theme ‘Communicative skills’ (7 quotations)

Most students reported they felt confident about communicating during history taking, physical examination and IV cannulation, stating they were able to build good and trusting relationships with their patient.

Nevertheless, video-based supervision was found to be helpful, as some aspects that were not perceived or addressed by the supervisor, were detected by the students themselves while watching the video. One student realised that she had ‘used her hands too much while talking, like some kind of sign language’ , concluding that she should ‘get rid of that habit’.

##### Theme ‘Procedural technical skills’ (13 quotations)

Generally, students reported they felt confident in the technique and framework of history taking. Some students ‘did not note making any mistakes’ , or ‘forgot [to ask] some things, but overall, … found it [history taking] to be pretty good’. One student thought he ‘knew the general sequence in history taking’ , but there were ‘specific gaps of knowledge’ regarding the patient’s suspected diagnosis. Another stated that ‘more preparation [reading the case history] beforehand’ would have been beneficial. Most students began with an open question for their patient and followed with more specific questions. One student observed that ‘sometimes, when you let the patient talk, you will find out what you want without having to dig deeper’.

#### Category 4: relevance of feedback (167 quotations)

##### Theme ‘Patient’s feedback’ (22 quotations)

The patient’s layperson perspective was seen as beneficial, highlighting ‘some aspects [the students] didn’t think of before’. One patient, for example, said they had no interest in being informed about the technical details of ECGs, when the student had not realised that they had given detailed information when performing the ECG. However, feedback from the actual patients was considered less beneficial than that from specifically trained actors. Although, one student stated: ‘To be able to perform these skills on actual patients [under supervision] is more valuable’. Nevertheless, feedback from patients was considered useful, but ‘not as useful as the supervisor’s feedback’.

##### Theme ‘Supervisor’s feedback’ (44 quotations)

The supervisor’s feedback was generally perceived as beneficial, especially in regard to procedural technical skills, as this provided a frank analysis from a different and expert perspective. One student thought that ‘generally, the feedback from the supervisor [was] more helpful [than video-based supervision]’. Overall, students preferred receiving the supervisor’s feedback before watching the video, as they could then focus their attention on important points: ‘I find the combination [that is the supervisor’s feedback plus video-based feedback] very helpful. The patient’s feedback is likely to bring less in the specific situations, but the supervisor’s feedback in combination with the video feedback is very helpful, I think’. Another student stated that sequential feedback was ‘helpful, especially as you got it [the supervisor’s feedback] right after [patient contact], and then watched the video, because then I realised that the feedback was really justified and it helped a lot, yes’.

##### Theme ‘Video-based feedback’ (101 quotations)

Video-based feedback was mostly positively received by students: ‘I self-reflect [upon my actions] a lot, but I experienced [my performance] in a different way thanks to the video’. However, most students felt it was only beneficial as an addition to the previous oral feedback from the supervisor. Sequential feedback was regarded as the most beneficial, that is first receiving feedback from the supervisor and then watching the video: ‘the video backs the supervisor’s feedback up, it is a tool for self-reflection, but without the supervisor’s oral feedback, it wouldn’t have helped much’. However, the video was not only considered beneficial for the evaluation of procedural technical skills, but also for communicative skills, with one student stating, ‘I usually do not see my body language’. Another student said, ‘sometimes, I cut my sentences off, finishing them half a minute later, with a long period of silence in between’, concluding that ‘[I] should speak more clearly and slowly, and let the patient finish her sentences’.

Not all aspects of the video-based supervision were seen positively. Some students felt uncertain about the benefit of filming procedures during on-ward supervision, feeling that the additional study of the video material ‘did not help that much because I do not know in which way it will influence my behaviour in future’; and ‘The problem is, I would not change anything [when performing the skill again] due to the video’.

In addition, students expressed a desire for the evaluation of more complex procedures through future video-based supervision, such as placing central venous catheters.

### Patients’ quantitative assessment

The patients’ ratings regarding their participation and subjective impressions are presented in Table 5. Overall, patients thought video-based, on-ward supervision was an important part of practical training for final year medical education. They were not discomforted by filming and did not feel that it falsified the situation. Although patients did not feel any added benefit from the supervision themselves, they generally agreed they would participate again in video-based, on-ward supervision in future.

**Table 5:** Patients’ (n=8) quantitative post-assessment to evaluate patients’ impressions of the session using a Likert scale from 1–6 (1 = I fully disagree, 6 = I fully agree). Median and Interquartile range

Question	Median	Interquartile range
I was ashamed to be filmed	1	1–1.25
I felt inhibited due to being filmed	1	1–1.25
I felt discomforted by being filmed	1	1–1
Video-taping falsified the situation	1	1–1
I consider watching the video helpful for practical training of final year students	6	6–6
I benefitted myself from video-based on-ward supervision	3,5	1.75–5
I would participate in video-based supervision again in the future	6	6–6

## Discussion

To our knowledge, the present pilot study is the first to describe an innovative approach for video-based, on-ward supervision. The implementation of this method in an on-ward setting was well accepted by both students and patients. Our analysis of the process of video-based, on-ward supervision and its determinants highlighted that participating students found sequential feedback most beneficial, as watching the video after hearing the supervisor’s feedback gave them opportunity to self-reflect on their performance. However, students considered the supervisor’s feedback the most beneficial element of supervision. In terms of feasibility, video-based, on-ward supervision was a viable supervision method. However, additional resources are required to implement video-based, on-ward supervision as part of the educational routine, such as acquiring the necessary equipment and personnel to manage and coordinate the process, thus ensuring a beneficial one-on-one learning experience.

On-ward supervision has already proven to be well accepted in preliminary work [17] and is also effective in improving ward round skills [53]. In the present study, video feedback was perceived to enhance teacher-directed feedback. In the perception of our participating students, video feedback only took full effect in connection with the feedback from the supervisor. As illustrated in our approach, video feedback can be used as a tool for modified, individualised feedback, meaning aspects to which the feedback should be tailored have to be defined prior to the training session [54]. In our case (in terms of modified, individualised feedback), the students’ focus when watching the video after the procedure was defined by the student and supervisor in a joint process. Our findings show that by viewing the video, added benefit can be achieved, especially in regard to non-verbal aspects (for example gestures, posture) and professional conduct. Moreover, our study indicates that video feedback can be a valuable addition to feedback from supervisors in a ward setting. Participants regarded the use of real patients in the supervision sequence as particularly valuable and relevant. Previous interventions with video feedback have mostly focused on the acquisition of communicative skills using standardised patients (SPs) [55]. Although even experienced doctors can hardly distinguish SPs from real patients in undercover operations [56], one student stated that SPs tend to give more detailed and beneficial feedback. Moreover, students also noted that feedback from real patients is often not as elaborate as feedback from a SP. Nevertheless, students appreciated the inside view of being able to work with real patients. Even if the importance of professional feedback from well-trained SPs is undisputed [57], access to feedback from real patients is of crucial importance for future doctors. Real patients may also be more eligible to evaluate professionalism, interpersonal and communication skills. We were surprised to find that the students preferred not to ask patients to participate themselves as they felt it would compromise the realism of the supervision. Moreover, the students reported that the chosen setting (the ward on which the students worked as the supervision site) was highly relevant, as it raised the level of realism. Although previous research (mostly drawing on simulations) has presented conflicting results regarding the influence of the degree of realism on the effect of learning, realism proved to be significant for the students in our study. Furthermore, they reported it was particularly important that the supervision was not disturbed. Surprisingly, the filming itself only played a minor role in terms of potential confounders.

Overall, the patients perceived the video-based supervision as valuable support for students. Although patients rarely reported personally benefiting from the supervision, they were happy to participate and did not perceive participation as a burden. This was an interesting finding, especially in view of the fact that little is known about the perceived burden for real patients participating in bedside teaching [58]. Finally, with regard to the costs of this innovative approach, our experiences with video-based, on-ward supervision showed no need for the presence of a second supervisor operating the camera, as a tripod can be used. Therefore, though a video-based approach might be more time-consuming (Table 1) than classic supervision models, it does not require additional personnel.

Some limitations of our study should be noted. Firstly, our study is limited by the small number of participants, possibly due to participation being voluntary, which may lead to biases in our analysis. As described by Schmidt and Bordage, the present study is a descriptive pilot study [59], which does not allow a statement on justification, although aspects requiring clarification were addressed in qualitative interviews. Therefore, the present study aimed to examine feasibility, obstacles to implementation and participants’ first impressions. However, the underlying supervision model of the present study has previously been proven to be effective in a controlled study of conducting ward rounds [53]. Further controlled studies are needed to investigate the extent of the benefits of video-based, on-ward feedback. A further limitation is the distribution of the performed activities in our study, with the majority of the students taking patient histories instead of performing practical or procedural skills, such as placing an IV cannula. However, as no statistical analysis was intended, we were able to collect a variety of impressions of procedural skills and their feasibility for video-based, on-ward supervision. Finally, although the qualitative analysis was performed according to grounded theory concepts and verified by a second analyst, our investigation should be regarded as a more subjective rather than a quantitative study. However, the openness in terms of assessment techniques and use of in-depth interviews and quotations enabled us to develop a comprehensive picture of this multi-layered topic.

## Conclusion

Our study found that video-based, on-ward supervision with individualised feedback was successful in improving the development of clinical skills of final year medical students. To determine the added value of filming patient interactions, future research should aim to objectively assess the added benefit to the supervision programme [17, 53] when integrating video-feedback, in terms of a justification study [59]. Moreover, it is unclear whether the benefits of video-based, on-ward supervision are higher for some skills or procedures than for others.
